# Non-polio enteroviruses in faeces of children diagnosed with acute flaccid paralysis in Nigeria

**DOI:** 10.1186/s12985-017-0846-x

**Published:** 2017-09-12

**Authors:** T. O. C. Faleye, M. O. Adewumi, M. O. Japhet, O. M. David, A. O. Oluyege, J. A. Adeniji, O. Famurewa

**Affiliations:** 1 Department of Microbiology, Faculty of Science, Ekiti State University, Ado-Ekiti, Ekiti State Nigeria; 2 Department of Virology, College of Medicine, University of Ibadan, Ibadan, Oyo State Nigeria; 3 Department of Microbiology, Faculty of Science, Obafemi Awolowo University, Ile-Ife, Osun State Nigeria; 4 WHO, National Polio Laboratory, Department of Virology, College of Medicine, University of Ibadan, Ibadan, Oyo State Nigeria; 5 Microbiology Unit, Department of Biological Sciences, Kings University, P.M.B. 555, Odeomu, Osogbo, Osun State Nigeria

**Keywords:** AFP, Enteroviruses, Nigeria, Non-polio enteroviruses, VP1 analysis

## Abstract

**Background:**

The need to investigate the contribution of non-polio enteroviruses to acute flaccid paralysis (AFP) cannot be over emphasized as we move towards a poliovirus free world. Hence, we aim to identify non-polio enteroviruses recovered from the faeces of children diagnosed with AFP in Nigeria.

**Methods:**

Ninety-six isolates, (95 unidentified and one previously confirmed Sabin poliovirus 3) recovered on RD cell culture from the stool of children <15 years old diagnosed with AFP in 2014 were analyzed. All isolates were subjected to RNA extraction, cDNA synthesis and three different PCR reactions (one panenterovirus 5′-UTR and two different VP1 amplification assays). VP1 amplicons were then sequenced and isolates identified.

**Results:**

92.71% (89/96) of the isolates were detected by at least one of the three assays as an enterovirus. Precisely, 79.17% (76/96), 6.25% (6/96), 7.30% (7/96) and 7.30% (7/96) of the isolates were positive for both, positive and negative, negative and positive, as well as negative for both the 5′-UTR and VP1 assays, respectively. In this study, sixty-nine (69) of the 83 VP1 amplicons sequenced were identified as 27 different enterovirus types. The most commonly detected were CV-B3 (10 isolates) and EV-B75 (5 isolates). Specifically, one, twenty-four and two of the enterovirus types identified in this study belong to EV-A, EV-B and EV-C respectively.

**Conclusions:**

This study reports the circulating strains of 27 non-polio enterovirus types in Nigerian children with AFP in 2014 and Nigerian strains of CV-B2, CV-B4, E17, EV-B80, EV-B73, EV-B97, EV-B93, EV-C99 and EV-A120 were reported for the first time. Furthermore, it shows that being positive for the 5′-UTR assay should not be the basis for subjecting isolates to the VP1 assays.

## Highlights

- Identification of circulating enterovirus types in Nigerian children with AFP.
- Sequence data documented for 27 different enterovirus types circulating in Nigeria.
- First report of CV-B2, CV-B4, E17, EV-B80, EV-B73, EV-B97, EV-B93, EV-C99 and EV-A120 in Nigeria.
- Positivity for 5′-UTR assay should not be the basis for subjecting enterovirus isolates to VP1 assays.
- Results suggest importation of enterovirus clades, especially from Asia into Nigeria.

## Background

Enteroviruses (EVs) are members of the genus *Enterovirus* in the family Picornaviridae*,* order Picornavirales. There are 13 species within the genus, four (EV-A to EV-D) of which (alongside the Rhinoviruses) have been repeatedly found to infect humans. The enterovirus capsid is a non-enveloped icosahedron with a diameter of 20–30 nM. It encloses a positive sense, single stranded RNA genome of ~7500 nt. The genome has untranslated regions (5′ and 3′ UTRs) flanking a single one open reading frame (ORF), whose polyprotein product is auto-catalytically cleaved into eleven proteins; four structural (VP1 – VP4) and seven non-structural (2A – 3D) proteins. Amplification of the 5′-UTR and/or the VP1 region can be used to detect the presence of enteroviruses [1–5], and the nucleotide sequence of the VP1 gene is used to identify enterovirus isolates [1–6].

Poliovirus, the best studied member of the genus *Enterovirus* and the etiologic agent of poliomyelitis belongs to species C within the genus. Humans remain the only known host of poliovirus, thus suggesting feasibility of its eradication. Consequently, in 1988, the World Health Assembly (WHA) resolved to eradicate poliomyelitis by the year 2000 [7], and the Global Polio Eradication Initiative (GPEI) was established. Courtesy of the GPEI’s activities, by year 2015 indigenous poliovirus transmission has been interrupted globally except in Pakistan and Afghanistan (www.polioeradication.org). This success has been the result of effective vaccination and intensive surveillance. Two poliovirus vaccine preparation (Oral Polio Vaccine [OPV] and Inactivated Polio Vaccine [IPV]) are currently being used by the GPEI and both are very effective [8]. However, due to the reversion of OPV, as part of the ‘end game’ strategy, the GPEI is tilting towards IPV as we approach the final phase of polio eradication [9].

Coupled with the vaccination effort is a very effective surveillance network that looks for polioviruses in both sewage-contaminated water (Environmental Surveillance [ES]) and children below the age of 15 years diagnosed with AFP. The ES strategy searches for enteroviruses in sewage-contaminated water due to the fact that all enterovirus infected individuals shed the virus in large amounts in faeces for several weeks and in turn into sewage and/or sewage contaminated water [10] . Therefore, ES is very sensitive and can detect enterovirus isolates from both symptomatic and asymptomatic individuals. The demerit of ES based strategy is that, alone, it cannot differentiate which isolates are associated with clinical manifestations and hospitalisations. On the other hand, AFP surveillance finds enteroviruses associated with a clinical manifestations and consequent hospitalisation. However, considering that AFP surveillance detects only the <10% of enterovirus infections with clinical manifestations, the inability of AFP surveillance to see beyond the tip of the iceberg is the strength of the ES surveillances strategy. Therefore, combining both strategies better illuminates our understanding of the epidemiological and evolutionary trajectory of enteroviruses, particularly with respect to pathogenicity. Hence, the reason the ES-AFP surveillance strategies are combined by the Global Polio Eradication initiative (GPEI) in some countries [11, 12].

As part of the surveillance network are about 150 laboratories globally (Global Polio Laboratory Network [GPLN]) that use the RD-L20B isolation protocol [11, 12] for poliovirus isolation. The RD-L20B isolation protocol is predicated on two different cell lines, RD (from a striated muscle cancer) [13] and L20B (a murine transgenic L cell that expresses the poliovirus receptor and is consequently permissive and susceptible to polioviruses) [14, 15]. As a by-product of the poliovirus surveillance programme, the GPLN recovers several non-polio enteroviruses (NPEVs) on the RD cell line.

The earliest molecular epidemiology study documenting NPEV from AFP cases in Nigeria was carried out using the RD-L20B protocol between 2002 and 2004 [16, 17]. Subsequent studies [18–22] generating nucleotide sequence data on enterovirus diversity in Nigeria have been largely ES based. The only exception is a recent study [23] on enterovirus diversity in healthy Nigerian children, in which a cell-culture independent enterovirus detection strategy was employed. Consequently, in this study we revisit NPEVs from AFP cases in Nigeria in an attempt to revise their diversity in individuals with this clinical condition in the region.

## Methods

### Samples

Ninety-six (96) RD cell culture isolates were analysed in this study. These isolates were recovered from the faeces of children below the age of 15 years that were diagnosed with AFP. A total of 8786 samples were received by the polio lab from January to June 2014, 7005 of which had no viral detection. Isolates were recovered from 1781 samples, 1024 of which were non-poliovirus isolates. Hence, the non-poliovirus isolates we attempted to characterize in this study represent 9.3% (95/1024) of those recovered by the polio lab during the period. The stool samples from which these isolates were recovered were collected in accordance with the national ethical guidelines as part of the National AFP surveillance programme in Nigeria and sent to the WHO National Polio Laboratory in Ibadan, Nigeria (subsequently referred to as the polio lab) to ascertain whether poliovirus is the etiologic agent of the diagnosed AFP using the WHO algorithm [12]. The isolates analyzed in this study were subsequently anonymized for further studies.

The WHO algorithm stipulates that fecal suspension from all AFP cases be inoculated into RD and L20B cell lines. Isolates that show cytopathic effect (CPE) on both cell lines are considered to be polioviruses, and subsequently subjected to intratypic differentiation (ITD) to differentiate between the wild type and the vaccine strain. On the other hand, isolates that only show CPE in RD cell line are assumed to be non-polio enteroviruses and stored away since they are not of ‘urgent’ programmatic importance to the GPEI.

To assemble the 96 isolates analyzed in this study, from the archives, sixteen (16) isolates that showed CPE in RD cell line only, were randomly selected each month from January to May 2014. In June, fifteen (15) isolates were selected, and a previously identified Sabin strain poliovirus 3 was added to serve as control for the study. Besides this known isolate, none of the other 95 isolates had been previously identified.

### RNA extraction and cDNA synthesis

**Fig. 1:**
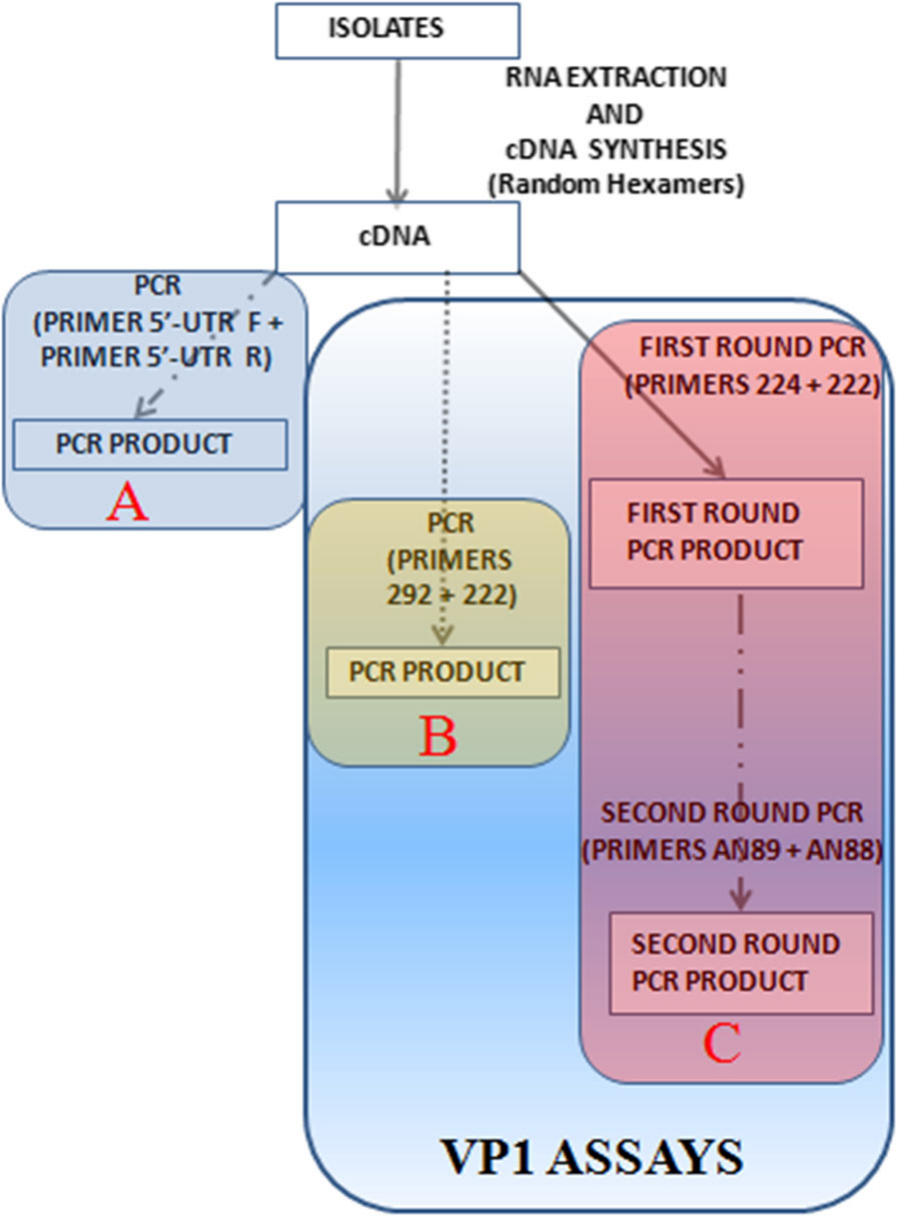
The algorithm used in this study. A depicts the 5′-UTR assay. B and C show the two VP1 assays. While B has only one stage of PCR, C has two consecutive stages (snPCR) of PCR

The algorithm followed in this study is as depicted in Fig. 1. Using the RNA extraction kit (Jena Bioscience, Jena, Germany), RNA was extracted from the isolates according to the manufacturer’s instructions. Script cDNA Synthesis Kit (Jena Bioscience, Jena, Germany) was used for complementary DNA (cDNA) synthesis as instructed by the manufacturer. Specifically, random hexamers were used for cDNA synthesis as previously described [20]. The cDNA was then stored at −80 °C and used for all polymerase chain reaction (PCR) assays.

### Polymerase chain reaction (PCR) assays

As shown in the algorithm for this study (Fig. 1), three different PCR assays were run; one 5′-UTR and two VP1 assays. The 5′-UTR and first PanEnterovirus VP1 PCR (PE-VP1-PCR) were one-step PCR assays. The second PanEnterovirus VP1 PCR was a semi-nested PCR assay (PE-VP1-snPCR) and was used to amplify the partial VP1 gene in those isolates for which the PE-VP1-PCR was negative. The 5′-UTR assay targets the 5’untranslated region which is very conserved but cannot be used to determine enterovirus type. The remaining two assays both target the highly variable VP1 region which is protein coding and encodes one of the capsid proteins. Unlike the 5’UTR, nucleotide sequence of the VP1 region can be and is used for enterovirus type determination [1–6]. It is essential to note, as mentioned above, that one of the VP1 assays is a one-step RT-PCR assay while the other is an RT- semi-nested PCR assay. Only the VP1 amplicons generated were sequenced and subsequently used for enterovirus type determination (Fig. 1).

All primers were made in 100 μM concentrations and all PCR assays were carried out in 30 μL reaction volumes. All PCR products were resolved on 2% agarose gels stained with ethidium bromide and viewed using a UV transilluminator.

#### 5′-UTR PCR and PanEnterovirus VP1 PCR (PE-VP1-PCR)

Primers panent 5′-UTR F and panent 5′-UTR R [12] were used for the 5′-UTR PCR assay while primers 229 and 222 [2, 6] were used for the PE-VP1-PCR assay. Each 30 μL reaction contained 6 μL of Red Load Taq (Jena Bioscience), 5 μL of cDNA, 0.3 μL of each primer and 18.4 μL of RNase-free water. Thermal cycling was done as follows: 94 °C for 3 min, 45 cycles of denaturation at 94 °C for 30 s, annealing at 42 °C for 30 s, and extension at 60 °C for 30 s with ramp of 40% from 42 °C to 60 °C. This was followed by 72 °C for 7 min, and thereafter the sample was held at 4 °C until the reaction was terminated.

#### PanEnterovirus VP1 semi-nested PCR (PE-VP1-snPCR)

This assay is a semi-nested PCR assay. Primers 224 and 222 [5, 6] were used for the first round PCR while primers AN89 and AN88 [5, 6] were used for the second round PCR assay. For the first round PCR assay, the 30 μL reaction contained 6 μL of Red Load Taq (Jena Bioscience), 5 μL of cDNA, 0.3 μL of each primer and 18.4 μL of RNase-free water. Thermal cycling was done as follows: 94 °C for 3 min, 45 cycles of denaturation at 94 °C for 30 s, annealing at 42 °C for 30 s, and extension at 60 °C for 60 s with ramp of 40% from 42 °C to 60 °C. This was followed by 72 °C for 7 min, and thereafter the sample was held at 4 °C until the reaction was terminated. The conditions were the same for the second round PCR assay except for following modifications: Instead of cDNA, the product of the first round PCR assay was used as template for the second round assay. Also, the extension time for the second round assay was 30 s as opposed to 60 s for the first round assay.

#### Amplicon sequencing and enterovirus typing

Only amplicon of positive PCR reactions for the two VP1 PCR assays (PE-VP1-PCR and PE-VP1-snPCR) were sequenced using the respective forward and reverse primers for each of the assays. Amplicons were shipped to Macrogen Inc., Seoul, South Korea, for purification and sequencing. Subsequently, enterovirus genotype and species were determined using the Enterovirus Genotyping Tool [24].

#### Phylogenetic analysis

The CLUSTAL W program in MEGA 5 software [25] was used with default settings to align sequences of four (the two most commonly isolated and the two for which most extensive nucleotide sequence data from the region exists) of the enterovirus serotype described in this study alongside those retrieved from GenBank. Subsequently, a neighbor-joining tree was constructed using the same MEGA 5 software with the Kimura-2 parameter model [26] and 1000 bootstrap replicates. The accession numbers of sequences retrieved from GenBank for this analysis are indicated in the sequence names on the phylograms.

#### Nucleotide sequence accession numbers

All sequences reported in this study have been deposited in GenBank and assigned accession numbers KX580638 – KX580702 and KX656918 – KX656912.

#### Ethics approval

For clarity sake it is crucial to re-iterate that enterovirus isolates were analysed in this study. The stool samples from which these isolates were recovered were collected in accordance with the national ethical guidelines as part of the National AFP surveillance programme in Nigeria and sent to the WHO National Polio Laboratory in Ibadan, Nigeria to ascertain whether poliovirus is the etiologic agent of the diagnosed AFP using the WHO algorithm [12]. The isolates analyzed were subsequently anonymized for further studies before use in this study. Thus, this article does not contain any studies with human participants performed by any of the authors. In addition, no information that can be used to associate the isolates analyzed in this study to any stool samples or the individual from which they were collected is included in this manuscript.

## Results

### 5′-UTR assay

Precisely, 85.42% (82/96) of the isolates analyzed, including the previously identified Sabin 3, had the expected ~114 bp band size and were consequently positive for the 5′-UTR PCR screen (Table 1).

**Table 1:** Summary of isolates characterized in this study. Where indicated, the asterisk (*) links the indicated amplicon to its enterovirus type, and ‘()’ denotes the number of amplicons that were successfully identified

S/N	Month	Isolates screened	Positive for 5′-UTR screen	Positive for VP1 screen	Positive for both 5′-UTR and VP1 screen	Positive for 5′-UTR and negative for VP1 screen	Negative for 5′-UTR and positive for VP1 screen	Negative for both 5′-UTR and VP1 screen	Total isolates identified	Serotypes (number of isolates)
1	January	16	14	14	13 (12)	1	1 (0)	1	12	CV-B3 (2), E7 (3), E13 (2), E19 (1), E20 (1), E33 (1), E6 (2)
2	February	16	14	15	13 (12)	1	2 (1)*	0	13	CV-B2 (1), CV-B3 (2), CV-B4 (1), E3 (2), E12 (1), E17 (1), E20 (3)*, E21 (1), EV-C99 (1)
3	March	16	14	13	13 (11)	1	0 (0)	2	11	CV-B3 (1), CV-B4 (1), CV-B5 (2), E11 (2), E12 (1), E13 (1), E30 (1), EV-B75 (1), EV-B80 (1)
4	April	16	13	14	12 (11)	1	2 (1)*	1	12	CV-B3 (3)*, E1 (2), E11 (1), E19 (1), E21 (1), EV-B73 (1), EV-B80 (2), EV-A120 (1)
5	May	16	13	12	11 (10)	2	1 (0)	2	10	CV-B3 (2), E6 (2), E13 (1), E14 (1), E19 (1), E26 (1), EV-B75 (2)
6	June	16	14	15	14 (11)	0	1 (0)	1	11	CV-B4 (1), CV-B5 (2), E7 (1), E11 (1), E19 (1), EV-B75 (2), EV-B93 (1), EV-B97, SPV3 (1)
	TOTAL	96	82	83	76 (67)	6	7 (2)	7	69	

### VP1 Assays (PE-VP1-PC and PE-VP1-snPCR)

Fifty-nine (59) of the samples analyzed in this study, excluding the previously identified Sabin 3, had the ~350 bp expected band size and were consequently positive for the PE-VP1-PCR screen. The remaining thirty-seven (37) samples were negative for the PE-VP1-PCR screen.

Of the remaining thirty-seven (37) samples, twenty-four (24), including the previously identified Sabin 3, had the ~350 bp expected band size and were thereby positive for the PE-VP1-snPCR screen. The remaining 13 samples were negative for the PE-VP1-snPCR screen.

Altogether, 86.46% (83/96) of the isolates, including the previously identified Sabin 3, had the expected ~350 bp band size and were consequently positive for the VP1 assays. The remaining 13.54% (13/96) were negative for the VP1 assays (Table 1).

### 5′-UTR versus VP1 assays

Overall, 92.71% (89/96) of the isolates were detected by at least one of the three assays as an enterovirus. Precisely, 79.17% (76/96) of the isolates were positive for both the 5′-UTR and VP1 assays. Furthermore, 6.25% (6/96) of the isolates were positive for the 5′-UTR assay alone, 7.30% (7/96) were positive for only VP1 assays and 7.30% (7/96) of the isolates were negative for both the 5′-UTR and VP1 assays (Table 1).

### Enterovirus typing

Sixty-nine (69) of the 83 amplicons generated from the VP1 PCR assays and successfully sequenced were identified. The remaining 14 could not be typed due to the presence of multiple peaks in their electropherograms. Twenty-seven different enterovirus types were identified from the 69 exploitable amplicons (Table 2). Specifically, one (1), twenty-four (24) and two (2) of the enterovirus types identified in this study belong to EV-A, EV-B and EV-C respectively (Table 2).

**Table 2:** Summary of enterovirus serotypes identified in this study

S/N	Types		Months detected	Number of isolates	Cumulative number
	EV-A				
1		EV-A120	Apr (1)	1	1
	EV-B				
2		CV-B2	Feb (1),	1	2
3		CV-B3	Jan (2), Feb (2), Mar (1), Apr (3), May (2)	10	12
4		CV-B4	Feb (1), Mar (1), Jun (1)	3	15
4		CV-B5	Mar (2), Jun (2)	4	19
6		E1	Apr (2)	2	21
7		E3	Feb (2)	2	23
8		E6	Jan (2), May (2)	4	27
9		E7	Jan (3), Jun (1)	4	31
10		E11	Mar (2), Apr (1), Jun (1)	4	35
11		E12	Feb (1), Mar (1)	2	37
12		E13	Jan (2), Mar (1), May (1)	4	41
13		E14	May (1)	1	42
14		E17	Feb (1)	1	43
15		E19	Jan (1), Apr (1), May (1), June (1)	4	47
16		E20	Jan (1), Feb (3)	4	51
17		E21	Feb (1), Apr (1)	2	53
18		E26	May (1)	1	54
19		E30	Mar (1)	1	55
20		E33	Jan (1)	1	56
21		EV-B73	Apr (1)	1	57
22		EV-B75	Mar (1), May (2), Jun (2)	5	62
23		EV-B80	Mar (1), Apr (2)	3	65
24		EV-B93	Jun (1)	1	66
25		EV-B97	Jun (1)	1	67
	EV-C				
26		EV-C99	Feb (1)	1	68
27		SPV3	Jun (1)	1	69

### Phylogenetic analysis

**Fig. 2:**
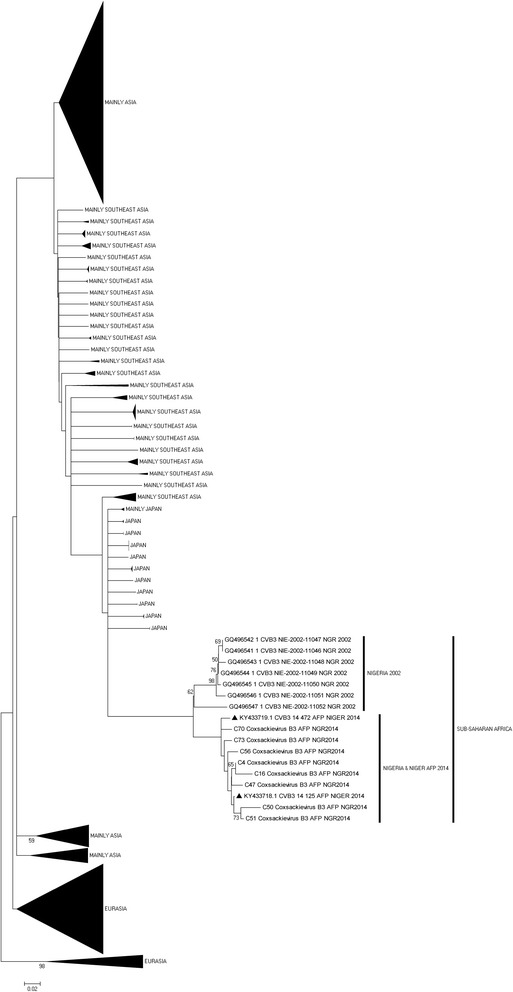
Phylogram of genetic relationship between VP1 nucleotide sequences of CV-B3 isolates. The phylogenetic tree is based on an alignment of the partial VP1 sequences. The CV-B3 sequences recovered in Nigeria in 2002 and the strains newly described in this study are indicated within sub-Saharan Africa (SSA). The strains indicated with black triangl represent CV-B3 strains from Niger; a country in west-Africa that shares a border with Northern-Nigeria

Of the different enterovirus types identified in this study, only four (Coxsackievirus B3 [CV-B3] and EV-B75 [the two most commonly isolated] and Echovirus 7 [E7] and E19 [the two for which most extensive nucleotide sequence data from the region exists]) were subjected to phylogenetic analysis. The CV-B3 tree (Fig. 2), showed that the lineage of the CV-B3 genotype detected in 2002 is different from the lineage detected in 2014. The CV-B3 isolates described in this study belong to the same lineage as those circulating in Niger; a country also in West-Africa which shares border with Nigeria in the North. In addition, it is important to note that the CV-B3 sequences detected in this study appear to share a distant common ancestor with those from Asia (Fig. 2).

**Fig. 3:**
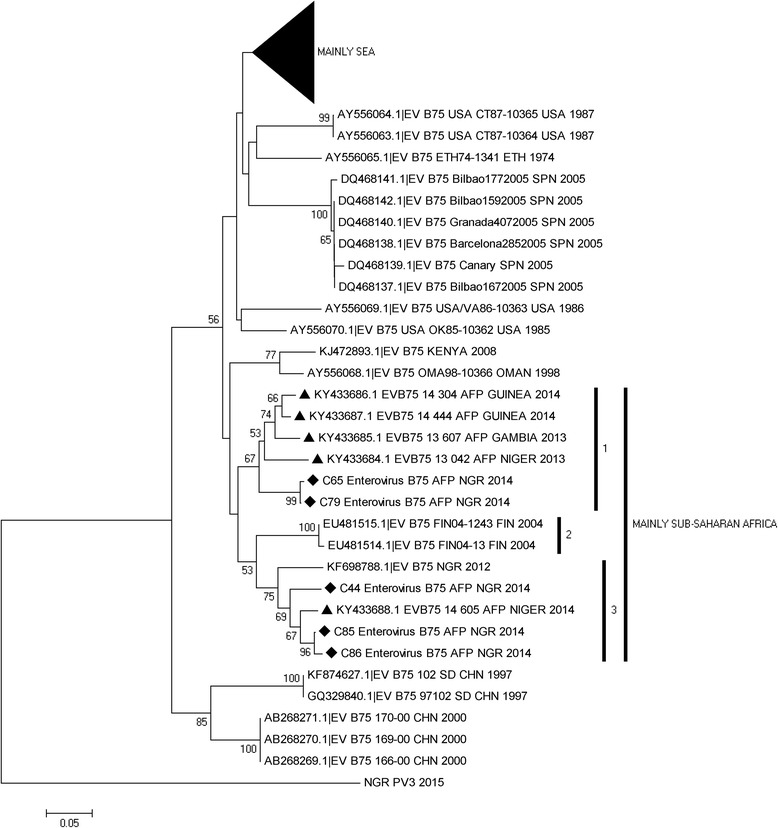
Phylogram of genetic relationship between VP1 nucleotide sequences of EV-B75 isolates. The phylogenetic tree is based on an alignment of the partial VP1 sequences. The newly sequenced strains are indicated with black diamond while other strains from west-Africa are indicated with black triangle. The GenBank accession numbers and strain of the isolates are indicated in the tree. Bootstrap values are indicated if >50%. SEA represents South-East Asia

For the EV-B75, two (sub lineages 1 and 3) different but closely related genotypes were detected. Both were however related to EV-B75 isolates detected in Finland in 2004 (sub lineage 2). Two of the EV-B75 isolates recovered in this study (sub lineage 1) share a common ancestor with a cluster found circulating in Niger, Gambia and Guinea. The other two EV-B75 isolates described in this study, (sub lineage 3) share a common ancestor with an isolate recovered from sewage contaminated water collected in Northern Nigeria in 2012 and an EV-B75 isolate recovered from a child with AFP in Niger (Fig. 3).

**Fig. 4:**
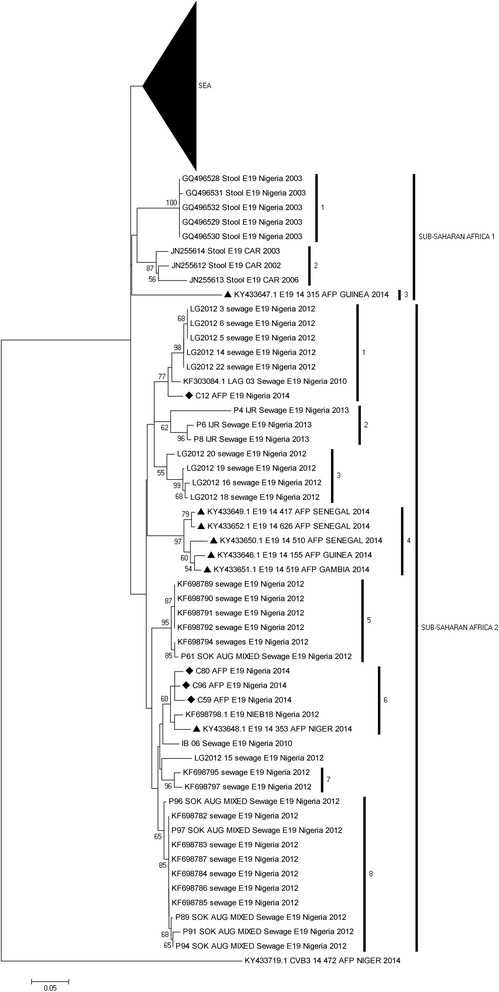
Phylogram of genetic relationship between VP1 nucleotide sequences of E19 isolates. The phylogenetic tree is based on an alignment of the partial VP1 sequences. The newly sequenced strains are indicated with black diamond while other strains from west-Africa are indicated with black triangle. The GenBank accession numbers and strain of the isolates are indicated in the tree. Bootstrap values are indicated if >50%. SEA represents South-East Asia

For the E19, two different genotypes were detected. Both were also very different from the E19 strains isolated in 2003 (SSA 1; sub lineage 1). The lineages to which the isolates belong are indicated as SSA 2; sub lineages 1 and 6 (Fig. 4). The single isolate from this study in SSA 2; sub lineage 1 shares a common ancestor with a lineage that was repeatedly isolated from sewage contaminated water in Lagos, Southwestern Nigeria since 2010. The remaining three strains however, belong to SSA 2; sub lineage 6 and are more closely related to an ES isolate recovered in Kano State, Northern Nigeria in 2012 and an isolate recovered from a child with AFP in Niger in 2014 (Fig. 4). The E19 isolates recovered from children with AFP in Gambia and Senegal belong to SSA 2; sub lineage 4 while that from Guinea belong to SSA 1; sub lineage 3.

**Fig. 5:**
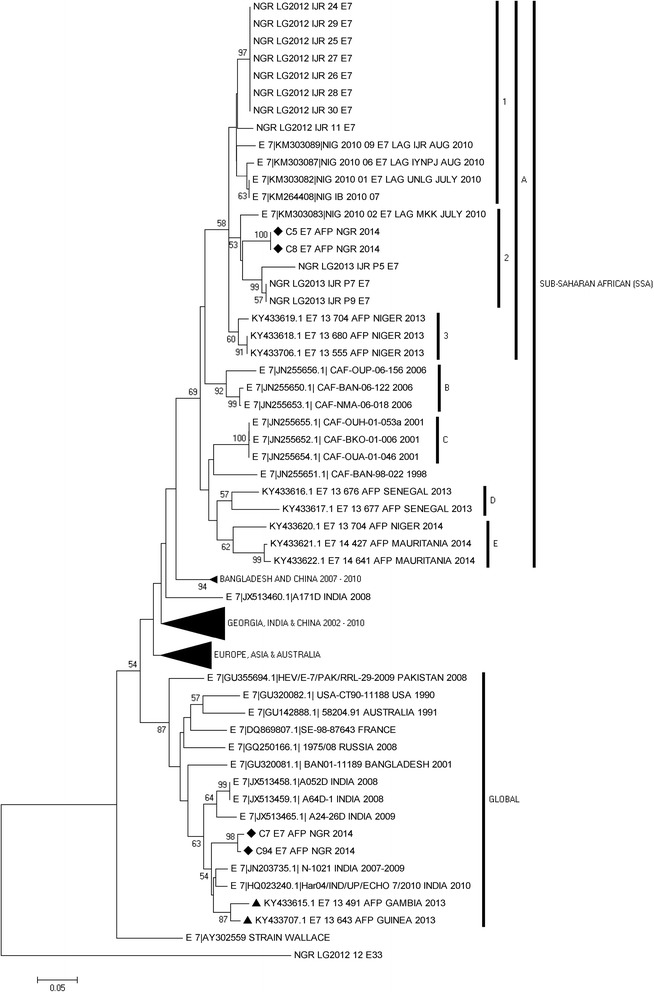
Phylogram of genetic relationship between VP1 nucleotide sequences of E7 isolates. The phylogenetic tree is based on an alignment of the partial VP1 sequences. The newly sequenced strains are indicated with black diamond while other strains from west-Africa within the Global cluster are indicated with black triangle. The GenBank accession numbers and strain of the isolates are indicated in the tree. Bootstrap values are indicated if >50%

As regards, E7, two different genotypes were also detected; SSA sub lineage A2 and the globally circulating lineage (Fig. 5). Two of the isolates from this study in SSA sub lineage A2 share a common ancestor with a lineage that was first isolated from sewage contaminated water in Lagos, Southwestern Nigeria in 2010. The remaining two strains however, belong to the global lineage and share a common ancestor with isolates repeatedly recovered from AFP cases in India since 2008 (Fig. 5).

## Discussion

In this study, we document nucleic acid sequence data for twenty-seven (27) different enterovirus types circulating and particularly present in children below the age of 15 years diagnosed with AFP in Nigeria in 2014. It is important to mention that prior this study, no nucleic acid sequence data existed in Genbank for nine (9) of these enterovirus types from Nigeria. To be precise, to the best of the authors’ knowledge, nucleic acid sequence data for Nigerian strains of E17, CV-B2, CV-B4, EV-B97, EV-B80, EV-B73, EV-B93, EV-C99 and EV-A120 are being reported for the first time. It is however, our opinion, that the fact that these molecular sequence data are being reported for the first time does not imply new introduction of these types into the region. Rather, we believe that these types had probably been around for a long time. Hence not detecting them might reflect the lack of interest in NPEVs because the global effort focused on eradicating polioviruses. However, as the goal of poliovirus eradication nears [9], there might be an upsurge of interest in NPEVs in a bid to better understand enterovirus biology and association with varying clinical conditions.

In this study, of the 69 isolates sequenced, 95.65% (66/69) belong to HEV-B representing 88.9% (24/27) of enterovirus types identified. (Table 2). This is consistent with the findings of other studies from the region [16, 17, 27] predicated on the RD-L20B algorithm, irrespective of whether healthy children [27] or those diagnosed with AFP were investigated [16, 17]. However, it was recently shown that when cell-culture bias is bypassed by the direct detection of enteroviruses from stool specimen, EV-As appear to be the most preponderant in the stool of healthy children from the region [23]. Furthermore, recent studies [20, 21] showed that most CV-A13s (EV-Cs) circulating in the region selectively replicate with evident cytopathology, and can be isolated on MCF-7, but not in RD cell line. Also, when the same sample was simultaneously inoculated into RD and MCF-7 cell lines, EV-Bs and EV-Cs respectively are specifically recovered on the two cell lines [21]. In fact, we recently observed [28] that when AFP samples that are negative for enteroviruses using the RD-L20B enterovirus detection algorithm [11] are subjected to the recently recommended [6] direct detection RT-snPCR algorithm described by Nix et al., [5], EV-Cs form the majority of enteroviruses detected. In addition, we also recently observed (unpublished) that about 50% of stool suspension from children with AFP in the region that yield EV-Bs in RD cell line also have an EV-C member present in the faecal suspension that was not detectable by the cell line. Put together, the preponderance of EV-B depicted by the results of this study and previous RD-L20B based studies from the region and globally should not be interpreted as an unbiased picture of the diversity landscape of enteroviruses in the AFP samples that yielded the isolates analyzed. Rather, it should be correctly viewed as the landscape as seen through the bias of RD cell culture.

Though in this study we describe enteroviruses present in the stool of children diagnosed with AFP, the findings of this study show the significance of merging ES and AFP data. Phylogenetic analysis (Figs. 3, 4 and 5) suggests that representatives of some of the lineages of EV-B75, E19 and E7 detected in the AFP cases had been previously detected through environmental surveillance. Interestingly, all the sequences from Nigeria in Figs. 4 and 5 except those from this study and the 2002–2004 sequences were from environmental surveillance. The ES data rightly show that more lineages are circulating in the population than depicted by the AFP data reported in this study. This thereby emphasizes the power of ES to illuminate our understanding of the diversity of enterovirus types and lineages circulating in the population. This will, consequently, further enable us to better understand the evolutionary trajectory of these enterovirus types and detect their silent circulation especially in the absence of clinical manifestations.

Surveillance of enterovirus diversity among healthy children can also increase the power of the ES-AFP surveillance strategy. For instance, it was previously shown [23] that other enterovirus types not detected in this study (e.g. EV-A71 and several CV-A types) were also present and circulating in Nigeria in the same year, 2014. In addition, we had shown [23] that the EV-B80 detected in a healthy Nigerian child belonged to the same lineage as those detected in children diagnosed with AFP in this study. We further showed [23] that the EV-C99 lineage found in a healthy Nigerian child is different from that described in this study. Thus, though not suggested or included in the GPEI surveillance algorithm, enterovirus surveillance in healthy children is useful and can significantly complement the ES-AFP strategy of the GPEI.

The baseline nucleotide sequence data for CV-B3 and E19 circulating in the region were generated in 2002 and 2003 respectively [16, 17]. Hence, the data presented in this study is a re-sampling of circulating strains of these types after over a 10-year period. For both types, the strains circulating when the baseline data was generated appear to have been completely replaced (Figs. 2 and 4). On the other hand, E7 and EV-B75 for which baseline sequence data from the region was generated in 2010 [18] and 2012 [22] respectively, members of the baseline lineage were still detected, however, alongside new lineages.

In some instances, it appears the variation in the population is seeded from another population. For example, as shown for CV-B3 and E7 (Figs. 2 and 5), it appears that in both instances, strains from Asia were imported into Nigeria and subsequently detected in the faeces of children diagnosed with AFP. These suspected importations corroborate what is known about poliovirus global circulation [8, 29]. What is not clear is why, as suggested in the regional confinement hypothesis [30], most non-polio enterovirus lineages detected in sub-Saharan Africa are yet to be detected and described in other world regions. Also interesting is the observation that enterovirus lineages found in Nigeria are also present and circulating in Niger (Figs. 2, 4 & 5). Considering the apparent porosity of borders in the Chad basin, this is not surprising. It is however crucial to note that the lineages circulating in Nigeria and Niger appear to be different from those recently shown [31] to be circulating in Gambia, Guinea and Senegal (Figs. 3, 4 & 5). Hence, though all the mentioned countries are in sub-Saharan Africa and more interestingly, West-Africa, it appears there might also be a level of sub-regional restriction of circulation. Characterization of more NPEV isolates will definitely show whether the observed is a true biological phenomenon or relic of paucity of data.

In this study, three (one 5′-UTR and two VP1) different enterovirus detection assays were used. The 5′-UTR screen is used to screen isolates in a bid to determine if they are enteroviruses. This is done because the 5’untranslated region (UTR) contains the internal ribosome entry site (IRES). On entry into the cytoplasm of any susceptible cell, to initiate translation of the single open reading frame (ORF) in the enterovirus genome, the ribosome must be assembled on the IRES in the 5′-UTR. The consequent conservation of this enterovirus genomic region is capitalized upon for enterovirus detection using the 5′-UTR assay. However, because of the conserved nature of the 5′-UTR its sequences do not provide the resolution needed to determine enterovirus type. On the other hand, the VP1 gene encodes one of the virus structural proteins. Studies [1–6] have established a correlation between the nucleotide or amino acid sequence of VP1 and enterovirus types determined using neutralization assays. The fact that there are over two hundred and fifty (250) enterovirus types (www.picornaviridae.com) gives a good impression of how variable the VP1 structural protein is. Hence, irrespective of how degenerate the primer combinations used, it might not be surprising if any one Panenterovirus RT-PCR screen fails to amplify the VP1 gene of any enterovirus of interest. Against this backdrop, enterovirus identification assays (including the poliovirus identification algorithm in use by the Global Polio Eradication Initiative [GPEI]) are built around first detecting the conserved enterovirus genomic region (5′-UTR) and subsequently subjecting isolates detected by this screen to VP1 assays [4–6].

Overall, six (6/96) of the isolates analyzed were positive for the 5′-UTR screen but negative for the VP1 screen. These isolates might therefore be enteroviruses that have VP1 primer binding sites that are too divergent to be bound by the primers used in this study. Another seven (7/96) isolates on the other hand were negative for the 5′-UTR screen but positive for the VP1 assays. Should we have used the 5′-UTR result as the basis for selecting isolates subjected to the VP1 screens, all these isolates would have been missed. It is currently not clear why these isolates were negative for the 5′-UTR screen considering they replicated in culture and are consequently viable strains. However, it is important to mention that recently, a new enterovirus 5′-UTR region was described that has a large deletion overlapping the region amplified in this assay [32–35]. Though described in EV-C isolates, it is not clear whether such constructs are present in members of other enterovirus species and whether such constructs would be functional when transferred to other species through recombination in the 5′-UTR region. Whatever be the case, the 5′-UTR primers used in this study could not detect these isolates. Consequently, the results of this study suggest that coupling the 5′-UTR and VP1 assays in a way that ensures both are independent and equally important for enterovirus identification might provide the added sensitivity required to find some divergent types. Particularly, it suggests that being positive for the 5′-UTR assay should not be the basis for subjecting isolates to the VP1 assays.

In this study, there were different groups of ‘untypable’ isolates (Table 1). The first group were those positive for the 5´-UTR screen but negative for the VP1 screen and have been addressed above. The second group were those positive for the VP1 assay but for which the eletropherogram could not be exploited due to multiple peaks. These are likely to come from cases where the children in question were co-infected with more than one type of enterovirus. This is not unusual and, as previously mentioned, we have more recently observed (unpublished) that in about 50% of cases, stool samples from children with AFP in Nigeria contain more than one enterovirus type and/or species.

The third group were those negative for both the 5′-UTR and the VP1 assays. Considering we detected isolates that were positive for the 5′-UTR screen but negative for the VP1 screen and vice-versa, it is not difficult to conceptualize the possibility that enterovirus isolates might exist that are negative for both assays. However, currently, this is only a conjecture. Since RD cell line can also support replication of other enteric viruses like the adenoviruses [36], these third group of ‘untypables’ might not necessarily be enteroviruses but could be other viruses for which the RD cell line is both susceptible and permissive.

## Conclusions

We identified 27 different enterovirus types present in Nigerian children with AFP in 2014 and document the first molecular detection/identification 9 Nigerian enterovirus strains, thereby expanding the catalogue of enterovirus types circulating and probably contributing to AFP in the region. There might also be importation into Nigeria of enterovirus clades from other world regions, and especially Asia. The study also shows that coupling the 5′-UTR and VP1 assays in a way that ensures both are independent and equally important for enterovirus identification might provide some of the added sensitivity required to detect more divergent enterovirus types. We therefore suggest revision of the current identification algorithm for enteroviruses and also surveillance of the environment, AFP and healthy children, as these have provided new information and may contribute significantly towards obtaining a complete picture of the diversity of enterovirus types present and circulating in any population.

## Abbreviations

AFP: Acute flaccid paralysis
cDNA: complementary Deoxyribonucleic Acid
CPE: Cytopathic effect
CV: Coxsackievirus
E: Echovirus
ES: Environmental surveillance
EV: Enterovirus
F: Forward
GPEI: Global Polio Eradication Initiative
GPLN: Global Polio Laboratory Network
IPV: Inactivated Polio Vaccine
L20B: Genetically engineered mouse cell line expressing the human cell surface receptor for poliovirus
MCF-7: Breast Cancer cell line
NPEV: Non-polio enterovirus
OPV: Oral Polio Vaccine
PCR: Polymerase chain reaction
PE: PanEnterovirus
R: Reverse
RD: Human rhabdomyosarcoma
RNA: Ribonucleic acid
UTR: Untranslated region
VP1: Virus Protein 1
WHA: World Health Assembly
WHO: World Health Organization
